# Clinical course and prognostic factors of childhood Takayasu’s arteritis: over 15-year comprehensive analysis of 101 patients

**DOI:** 10.1186/s13075-018-1790-x

**Published:** 2019-01-22

**Authors:** Luyun Fan, Huimin Zhang, Jun Cai, Lirui Yang, Bin Liu, Dongmei Wei, Jiachen Yu, Jiali Fan, Lei Song, Wenjun Ma, Xianliang Zhou, Haiying Wu, Ying Lou

**Affiliations:** 1 State Key Laboratory of Cardiovascular Disease, National Center for Cardiovascular Diseases, Fuwai Hospital, Chinese Academy of Medical Sciences and Peking Union Medical College, Beijing, China; 2 Department of Cardiology, Beijing Anzhen Hospital, Capital Medical University, Beijing, China; 3 School of Basic Medicine, Chinese Academy of Medical Sciences and Peking Union Medical College, Beijing, China

**Keywords:** Takayasu arteritis, Children, Prognostic factor

## Abstract

**Background:**

Childhood Takayasu’s arteritis (c-TA) is scarcely reported but is characterized by devastating morbidity and mortality. This study aims to investigate the clinical course of c-TA and prognostic factors associated with rehospitalization and events including vascular complications, flares, and death.

**Methods:**

An ambispective study of 101 c-TA patients satisfying the American College of Rheumatology (ACR) criteria and/or the European League against Rheumatism (EULAR)/Pediatric Rheumatology International Trials Organization (PRINTO)/Pediatric Rheumatology European Society (PReS) criteria was conducted from January 2002 to December 2017. Data on demographic, clinical, laboratory, imaging, and therapeutic features were collected. Event-free survival, complication-free survival, flare-free survival, rehospitalization-free survival, and associated prognostic factors were assessed by Kaplan-Meier survival curve and propensity score analysis.

**Results:**

The median age at c-TA onset was 14 (interquartile range (IQR) 12–16) years and 76.2% were female. Hypertension (70.3%), blood pressure discrepancy (55.4%), bruits (51.5%), and pulse deficits (37.6%) were core presentations. Major vascular involvement included the renal artery (62.4%), abdominal aorta (42.6%), subclavian artery (43.6%), and carotid artery (42.6%). Glucocorticoids (78.2%), antihypertensive drugs (72.3%), antiplatelet agents (72.3%), and revascularization (57.4%) were made up the majority administered. At a median 2.4 (IQR 0.7–6.1) years of follow-up, events, rehospitalization, vascular complications, flares and death were observed in 44.6%, 37.6%, 44.6%, 26.7%, and 3%, respectively. The 5-year event-free survival, rehospitalization-free survival, vascular complication-free survival, and flare-free survival were 42.8%, 55.8%, 45.9%, and 62.3%, respectively. Body mass index (BMI) (hazard ratio (HR) = 0.49, 95% confidence interval (CI) 0.30–0.81, *p* = 0.005), stroke (HR = 7.37, 95% CI 2.35–23.1, *p* = 0.001), and revascularization (HR = 0.51, 95% CI 0.27–0.94, *p* = 0.032) were independent prognostic predictors of events. Predictors for rehospitalization include age at admission (HR = 0.81, 95% CI 0.69–0.94, *p* = 0.006), renal artery involvement (HR = 0.49, 95% CI 0.25–0.96, *p* = 0.037), and elevated C-reactive protein (CRP; HR = 2.50, 95% CI 1.24–5.00, *p* = 0.01). BMI level (*p* = 0.024) and renal artery involvement (*p* = 0.015) were also associated with vascular complications, while revascularization (*p* = 0.002) independently correlated with re-flares.

**Conclusions:**

This large ambispective study of c-TA revealed an early 3% mortality at the first year and around 50% morbidity within 5 years after diagnosis. Hypertension, renal artery involvement, and revascularization based on anti-inflammation, antihypertension, and antiplatelet medications dominated c-TA with indications for optimistic prognosis. Patients with initial lower BMI level, a younger age at admission, stroke, and elevated CRP have a high risk of poor outcomes, requiring close c-TA monitoring and more aggressive management.

**Trial registration:**

NCT03199183, unique protocol ID: 2016-ZX43. June 26, 2017

**Electronic supplementary material:**

The online version of this article (10.1186/s13075-018-1790-x) contains supplementary material, which is available to authorized users.

## Background

Takayasu’s arteritis (TA) is a chronic progressive vasculitis of unknown etiology, predominantly affecting the aorta and its primary branches in women aged from 20 to 40 years. The estimated incidence of TA in Europe and the USA is 0.4–2.6 per million people, while the prevalence is estimated to be over 0.003% in an Asian country [1, 2]. Other than early-stage constitutional symptoms, vascular complications such as stroke, myocardial infarction, resistant hypertension, and heart failure can emerge after arterial wall thickening, stenosis, occlusion, aneurysm, or thrombosis formation due to progressive inflammation. Hence, early inflammation control and vascular injury relief are the crucial goals of TA management. First-line therapy is glucocorticoids (GCs), with additional immunosuppressant and biological agents increasingly administered for GC-resistant TA or GC-sparing facilitation, while surgery or interventions are still necessary for end-organ/limb ischemia amelioration and the near 20% of TA patients without response to any medications [2, 3].

Childhood TA (c-TA), the most common large vessel vasculitis in the pediatric population, has been studied in 17 cohorts of 445 c-TA cases (including 24 Chinese patients) to date, with an uncertain clinical profile but devastating mortality reaching 27% (Additional file 1: Table S1) [4–9]. Notably, comprehensive data on management and outcome prediction of c-TA are extremely lacking. To address these issues, the present study aims to: 1) describe the clinical phenotypes, angiographic findings, and diagnostic algorithm of c-TA; 2) summarize the therapeutic strategies and prognosis for c-TA; and 3) identify the predictors of c-TA outcomes, including vascular complications, flares, all-cause death, and rehospitalization.

## Methods

### Patients and inclusion criteria

We conducted an ambispective cohort study of 101 c-TA patients in Fuwai Hospital, Chinese Academy of Medical Sciences, with 96 patients retrospectively recruited from January 2002 to December 2016 and five patients prospectively enrolled from January 2017 to December 2017. A total of 99 cases hospitalized under 18 years of age were enrolled, fulfilling at least three of the 1990 American College of Rheumatology (ACR) criteria [10] or the 2010 European League against Rheumatism (EULAR)/Pediatric Rheumatology International Trials Organization (PRINTO)/Pediatric Rheumatology European Society (PReS) criteria [11]. The 1990 ACR criteria (69 patients, 68.3%) included: 1) age at disease onset ≤ 40 years; 2) limb claudication; 3) pulse deficits over the brachial arteries; 4) blood pressure asymmetry between arms; 5) bruits over the subclavian arteries or aorta; and 6) angiographic abnormalities [10]. The EULAR/PRINTO/PReS criteria (99 patients, 98%) contained one major criteria of typical angiographic abnormalities, and at least one of following minor criteria: 1) pulse deficits or claudication; 2) blood pressure discrepancy in any limb; 3) bruits; 4) pediatric hypertension (defined as equal to or greater than the 95th percentile of children at the same age, sex, and height); and 5) elevated acute phase reactant (APR; increased erythrocyte sedimentation rate (ESR) ≥ 20 mm/h and/or C-reactive protein (CRP) ≥ 6 mg/L) [11]. Three patients not satisfying the ACR criteria or the EULAR/PRINTO/PReS criteria were included with a clinical diagnosis of c-TA. Case 1 presented angina pectoris with right coronary artery stenosis and whole aorta wall thickening proven by computed tomographic angiography (CTA). Case 2 complained of syncope and dyspnea with bilateral subclavian artery and coronary artery stenosis confirmed by CTA and coronary artery angiography. Case 3 presented dyspnea, cough, weight loss, and bruits over the pulmonary arteries. CTA revealed multiple pulmonary arterial stenosis, occlusion, and aneurysm formation. New-onset left pulmonary artery trunk stenosis and right upper pulmonary artery occlusion were observed at 1-year follow-up. One patient initially fulfilling the EULAR/PRINTO/PReS criteria (renal artery stenosis and pediatric hypertension) was excluded over a 10-year follow-up for fibromuscular dysplasia (FMD) diagnosis after a clinical panel consultation. This study was approved by the local ethics committee.

### Imaging modalities

Angioplasty was performed in all c-TA patients, including conventional angiography, CTA, and/or magnet resonance angiography (MRA) as the initial diagnostic modality in 36 (35.6%), 57 (56.4%), and 9 (8.9%), respectively, while 28 (27.7%) patients experienced additional catheter-based angiography for interventional therapy after TA diagnosis by CTA (24.7%, *n* = 25) and/or MRA (4%, *n* = 4). Catheter-based angiography was generally opted for based on clinical utility, the social-economic background, and our limited resources before 2007. After 2007, CTA and three-dimensional reconstruction of the whole aorta tree was increasingly utilized as the prime screening and diagnostic modality in our center for its high diagnostic accuracy and enabling visualization of vessel wall and luminal changes, while MRA remained little used due to the long turnaround time of evaluation in our institute.

### Therapy protocol

The core principles of therapy were inflammation remission and vascular injury relief/prevention. Patients with active disease were generally prescribed with prednisolone 0.5–1 mg/kg/day for at least 1 month based on individual disease activity, clinical experience, and limited resources in our institute. After inflammation control, GCs were gradually tapered (1.25–2.5 mg/day per month) to a maintenance dosage of 5–10 mg/day for at least half a year. Additional immunosuppressive agents after panel discussion and patient communication were initiated for GC-resistant TA or GC-sparing facilitation, particularly in patients with severe GC-relevant side effects. Mycophenolate mofetil (MMF) has been the first choice in our center with an initial dose of 0.5 g twice per day (bid) increased to 1 g bid at the third week under conditions of a normal blood test outcome. Methotrexate (10–15 mg/week), leflunomide (20–30 mg/day), or cyclophosphamide (preferred in the elderly male population, 0.2 g every other day) were the optional choice in those who cannot afford MMF. No biologic agents were prescribed in the childhood population due to sparse clinical trial evidence.

Revascularizations were performed in patients meeting the following indications: 1) symptomatic end-organ/limb ischemia or severe hypertension (systolic blood pressure (SBP) ≥ 160 mmHg and/or diastolic blood pressure (DBP) ≥ 100 mmHg, or SBP ≥ 140 mmHg and/or DBP ≥ 90 mmHg under three types of antihypertensive drugs including diuretics); 2) imaging evidence of vascular stenosis ≥ 70%; and 3) complete inflammation control except for life-threatening conditions requiring immediate interventions [12–15]. Endovascular angioplasty under close inflammation monitoring was preferred in children for further vascular growth and a lower risk of restenosis and re-intervention [12]. Stent placement was an alternate procedure when angioplasty failed, or for intervention performed in middle aortic stenosis [13, 14]. Open surgical procedures were rarely performed for their invasive nature and were adopted in patients with valvular involvement, aneurysm, or those not amenable to interventions.

Among patients undergoing revascularization, prednisone 0.25–0.5 mg/kg/day was prescribed at least 2 months prior to intervention and gradually reduced to 5–10 mg/day for at least 6 months after procedures, even in patients at a clinical quiescent stage. Antiplatelet drugs were also used in patients experiencing revascularizations (aspirin 100 mg/day 3 days before and 6 months after procedures, or aspirin 100 mg/day plus clopidogrel 75 mg/day 2 days before and 1 month after procedures) or at high risk of thrombosis for secondary prevention.

### Data collection

In our hospital, medical records were documented in a prospective manner with scanned documents of handwritten medical records (January from 2002 to December 2006) and electronic medical records (January 2007 onwards) available. We retrospectively reviewed medical records of all c-TA patients consecutively hospitalized from January 2002 to December 2016 and abstracted data from hospitalization and discharge summaries, doctor’s notes, outpatient medical reports, and examination reports (particularly vascular imaging). For the prospective cohort, data on demographic, clinical, laboratory, and imaging features were prospectively recorded from the first hospitalization of patients to each follow-up clinical visit or rehospitalization at 3, 6, and 12 months after discharge. Demographic data included age at TA onset and admission, sex, years of delay to diagnosis, and cardiovascular risk factors. The clinical profile consisted of constitutional symptoms, end-organ/limb ischemic presentations, laboratory results (ESR and CRP), and imaging features (affected vascular beds and type of lesions including stenosis (≥ 50% lumen), narrowing, aneurysm (diameter ≥ twofold lumen diameter), dilation, occlusion, dissection, and vessel wall thickening). The anatomical classification was based on the criteria of Hata et al. [16]: 1) type I, involving major branches of the aortic arch; 2) type IIa, affecting the ascending aorta, the aortic arch, and its branches; 3) type IIb, involving the ascending aorta, aortic arch and/or its branches, and the thoracic descending aorta; 4) type III, involving the thoracic descending aorta, the abdominal aorta, and/or the renal arteries; 5) type IV, affecting the abdominal aorta or renal arteries; and 6) type V, presenting the combined features of type IIb and type IV. Disease activity at each visit was assessed by the US National Institutes of Health (NIH) criteria [17]: 1) systemic symptoms; 2) raised APR; 3) new presentations of vascular ischemia or inflammation, such as claudication, pulse deficits, bruits, carotidynia, blood pressure discrepancy in four limbs; and 4) new or worsening angiographic lesions. The active disease phase was identified satisfying at least two NIH criteria. Current medications and interventional and surgical procedures were also recorded.

### Definition of endpoints

The primary endpoint was defined as the first occurrence of at least one event, a composite endpoint as previously described that consisted of vascular complications, disease flares, and all-cause death [18]. Vascular complication was defined as the occurrence of new-onset arterial occlusion or progressive stenosis requiring revascularization, new or worsening arterial aneurysm, new or progressive aortic regurgitation, new-onset myocardial infarction, new-onset or progressive heart failure, new-onset stroke or transient ischemic attack, or end-stage renal failure. Disease flare was identified as the recurrence of inflammation based on NIH criteria or necessitating altered treatment. A secondary outcome was identified as rehospitalization.

### Statistical analysis

Categorical data are presented as frequency and percentage. Continuous variables are reported as the mean ± standard deviation or median and interquartile range (IQR), depending on distribution. An independent *t* test or Mann-Whitney *U* test, chi-squared test or Fisher’s exact test were performed as appropriate for intergroup comparison of features of c-TA patients enrolled prior to and after 2007, referring to our clinic experience. Survival functions were estimated through Kaplan-Meier survival curves. Event-free survival was defined as the time from c-TA diagnosis to the first vascular complication, the first re-flare, all-cause death, or last follow-up. Rehospitalization-free survival was defined as the time from c-TA diagnosis to the first rehospitalization, death, or last follow-up. Complication-free survival/flare-free survival was defined as the time from c-TA diagnosis to the first vascular complication/first disease flare, death, or last visit. Predictors of outcomes were identified using Cox regression analysis, described with hazard ratios (HR) and 95% confidence intervals (CIs). Significant candidates for multivariate analysis consisted of complete variables with a *p* value < 0.05 in the univariate Cox model, and potential confounding factors including sex, age at admission, and body mass index (BMI). A bidirectional stepwise selection algorithm was performed in the multivariate model. The proportional hazards assumption was assessed by the Schoenfeld’s residuals test. All statistical analyses were performed at a two-sided significance level of 0.05 using SAS software version 9.4 (SAS Institute, Cary, North Carolina).

## Results

### Demographic characteristics and clinical manifestations

A total of 101 children with TA (76.2% female) were enrolled with an age at onset of 14 (IQR 12–16) years and delay to diagnosis of 0.6 (IQR 0.2–2.1) years. Over 1 year and 4 years of delay were observed in 46 (45.5%) and 15 (14.9%) patients, respectively. The median BMI was 19.7 (IQR 17.6–21.4) kg/m^2^ with 40.6% under 18.5 kg/m^2^ and 10.9% overweight (≥ 24 kg/m^2^). The most common presenting feature was cardiovascular manifestation, including arterial hypertension (70.3%), blood pressure discrepancy (55.4%), vascular bruits (51.5%), pulse weakness (37.6%), heart failure (24.8%), claudication (22.8%), and myocardial ischemia (3%), followed by neurological/ocular (44.6%) and pulmonary (33.7%) manifestations, and constitutional symptoms (29.7%). Tobacco use, hyperlipidemia, and diabetes mellitus were rarely observed in 1, 2, and 1 patients, respectively, with four diagnosed with tuberculosis prior to TA. The median ESR and CRP values were 10 (IQR 4–31) mm/h and 5.4 (IQR 1.6–15.4) mg/L, with elevated ESR in 32.7% and elevated CRP in 34.7%. Details of demographic and clinical features are depicted in Table 1 and Additional file 1 (Table S2).

**Table 1:** Demographic, clinical, and therapeutic features of childhood Takayasu’s arteritis (c-TA)

	Median/*n*	IQR/%
Demographic features		
Age at Takayasu’s arteritis onset, years	14	12–16
Delay to diagnosis/admission, years	0.6	0.2–2.1
BMI (kg/m^2^)	19.7	17.6–21.4
Sex (female)	77	76.2
Clinical presentations		
Hypertension	71	70.3
Blood pressure discrepancy	56	55.4
Bruits	52	51.5
Pulse deficits	38	37.6
Dyspnea	30	29.7
Dizziness	27	26.7
Heart failure	25	24.8
Claudication	23	22.8
Fever	13	12.9
Syncope	10	9.9
Stroke	6	5.9
Carotidynia	4	4
Myocardial infarction/ischemia	3	3
Retinopathy	38	37.6
Direct inflammation involvement	20	19.8
Secondary to hypertension	17	16.8
Angiographic classification		
Type I	12	11.9
Type IIa	0	0
Type IIb	10	9.9
Type III	9	8.9
Type IV	38	37.6
Type V	32	31.6
Laboratory results		
Elevated ESR	33	32.7
Elevated CRP	35	34.7
Medications		
Glucocorticoids	79	78.2
Immunosuppressant	11	10.9
Antiplatelet drugs	73	72.3
Antihypertensive drugs	73	72.3
Revascularization	58	57.4
Balloon angioplasty	41	40.6
Stenting	22	21.8
Surgery	8	7.9

*BMI* body mass index, *CRP* C-reactive protein, *ESR* erythrocyte sedimentation rate, *IQR* interquartile range

### Angiographic findings

The involved segments of the aorta included the abdominal aorta (42.6%), thoracic descending aorta (32.7%), aortic arch (16.8%), and ascending aorta (12.9%). Renal arteries (62.4%), subclavian arteries (43.6%), and carotid arteries (42.6%) were the major aorta branches involved. Pulmonary artery involvement, coronary artery involvement, and aortic regurgitation were confirmed in 11.9%, 5%, and 13.9%, respectively. Local lesions in the abdominal aorta/renal arteries (type IV according to the classification of Hata et al. [16], 37.6%) were the most common angiographic type, followed by type V (31.7%), type I (11.9%), type IIb (9.9%), and type III (8.9%) diseases. Stenosis and occlusion were the most popular lesions observed in 91.1% and 63.4%, respectively, while vessel wall thickening, narrowing, dilation, aneurysm, and dissection were detected in 28.7%, 16.8%, 16.8%, 11.9%, and 1%, respectively. Seven patients suspected of having vessel wall inflammation in our cohort received ^18^F-fluorodeoxyglucose positron emission tomography (^18^F-FDG-PET)/CT with a mean maximum standard uptake value (SUVmax) of 2.8 ± 0.8. Table 2 shows the frequencies of arterial involvement.

**Table 2:** Frequencies of arterial involvement and relevant interventions

	Patients (%) with arterial involvement	Patients (%) with interventions	Lesions (%) with interventions
Ascending aorta	13 (12.9%)	0 (0%)	0 (0%)
Aortic arch	17 (16.8%)	0 (0%)	0 (0%)
Thoracic aorta	33 (32.7%)	7 (6.9%)	7 (6.9%)
Abdominal aorta	43 (42.6%)	3 (3%)	3 (2.9%)
Carotid artery	43 (42.6%)	5 (5%)	4 (4.9%)
Subclavian artery	44 (43.6%)	10 (8.9%)	12 (11.8%)
Vertebral artery	14 (13.9%)	1 (1%)	1 (1%)
Renal artery	63 (62.4%)	42 (41.6%)	57 (55.9%)
Celiac trunk	17 (16.8%)	0 (0%)	0 (0%)
Superior mesenteric artery	14 (13.9%)	0 (0%)	0 (0%)
Iliac artery	13 (12.9%)	3 (3%)	3 (2.9%)
Pulmonary artery	12 (11.9%)	1 (1%)	1 (1%)
Coronary artery	5 (5%)	1 (1%)	1 (1%)

### Medication and revascularization

Immunosuppressive therapy was prescribed in 80 patients. GCs were administrated in 78.2% with a median initial dosage of 0.5 (IQR 0.4–0.6) mg/kg/day, while immunosuppressants were additionally prescribed in 10.9% with MMF, leflunomide, methotrexate, and cyclophosphamide in 5, 2, 3, and 2 patients, respectively. Only one patient administered with bosentan was without GCs for isolated pulmonary arteritis. Sixty-one (60.4%) hypertensive patients required antihypertensive agents median, 2 kinds (IQR 2–3), including calcium channel blocker (42.6%), β-blocker (34.7%), angiotensin-converting enzyme inhibitors/angiotensin receptor blocker (19.8%), and thiazides (4%). Seventy-three (72.3%) patients received antiplatelet agents with aspirin 100 mg/day in 49 patients and dual antiplatelet therapy in 24 patients. Furthermore, anticoagulants and statins were prescribed in 5.9% and 5%, respectively.

Fifty-eight (57.4%) patients underwent revascularization for 102 lesions: balloon angioplasty in 71 lesions in 41 patients, stenting in 23 lesions in 22 patients, three bypass grafts (one from the right axillary artery to the femoral artery, one from the right axillary artery to the left iliac artery, and one from the ascending aorta to the right common carotid artery), two aortic valve replacements for severe aortic regurgitation, one mitral valve replacement for infective endocarditis, one coronary artery bypass graft (CABG), and one right femoral artery pseudoaneurysm (a complication of intervention) resection. Renal arteries, subclavian arteries, the mid-aorta, and carotid arteries were the major vessels enduring endovascular interventions. Details of the therapies are depicted in Tables 1 and 2.

### Outcomes and prognostic factors

**Fig. 1:**
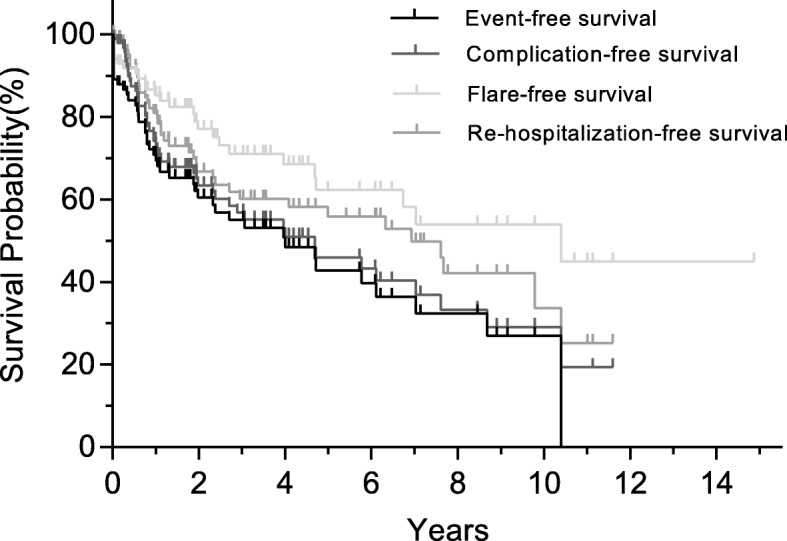
Event-free survival, complication-free survival, flare-free survival, and rehospitalization-free survival

**Fig. 2:**
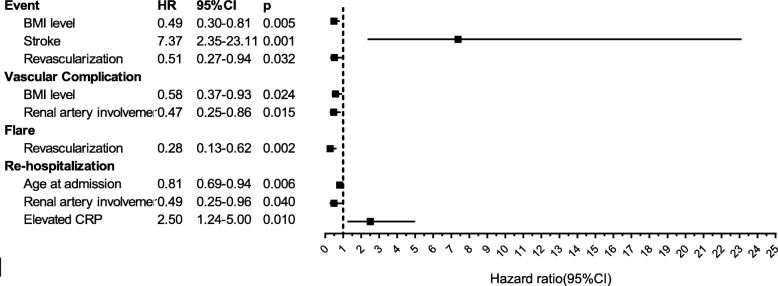
Prognostic factors of c-TA in multivariable Cox regression analysis. BMI body mass index, CI confidence interval, CRP C-reactive protein, HR hazard ratio

The median follow-up duration was 2.4 (IQR 0.7–6.1) years with 68, 45, 26, and 6 c-TA patients followed up over 1 year, 3 years, 5 years, and 10 years, respectively. Three patients died, two from recurrent acute heart failure due to severe and extensive mid-aortic stenosis when waiting for revascularization after inflammation control [14], and one with left descending coronary artery occlusion, severe right coronary artery stenosis (80–90%), and severe left circumflex artery stenosis (50–90%) without an opportunity for timely intervention. The overall survival rate was 96.1% at the first year and remained the same over the following period. Details of outcomes and outcome-free survival curves are depicted in Table 3 and Fig. 1. Outcome-associated prognostic factors are presented in Table 4 and Fig. 2.

**Table 3:** Long-term outcomes of childhood Takayasu’s arteritis (c-TA) patients

Outcomes	Number	Percent
Event	45	44.6
Rehospitalization	38	37.6
Death	3	3
Heart failure due to mid-aortic stenosis	2	2
Myocardial infarction	1	1
Vascular complication	45	44.6
New occlusion or progressive stenosis requiring revascularization	37	36.6
New-onset myocardial infarction	2	2
New/progressive heart failure	5	5
New/worsening aortic regurgitation	1	1
New/worsening aneurysm	8	7.9
New-onset stroke/transient ischemic attack	1	1
End-stage renal failure	0	0
Flare	27	26.7
New vascular ischemia or inflammation	17	16.8
New angiographic lesions	49	48.5
Systematic symptoms	6	5.9
Raised acute-phase reactant	17	16.8
Duration to outcomes, years	(Median)	(Interquartile range)
Time to the first event	1.04	0.01–3.53
Time to the first rehospitalization	1.69	0.57–4.85
Time to the first flare or death	1.69	0.31–4.26
Time to the first complication or death	1.45	0.41–4.04

**Table 4:** Prognostic factors of childhood Takayasu’s arteritis (c-TA) in univariable Cox regression analysis

Factors	Event		Vascular complication		Flare		Rehospitalization	
	HR (95% CI)	*p*	HR (95% CI)	*p*	HR (95% CI)	*p*	HR (95% CI)	*p*
BMI	0.50 (0.31–0.82)	**0.006**	0.56 (0.35–0.89)	**0.015**	0.44 (0.23–0.84)	**0.01**	0.83 (0.51–1.34)	0.44
Age at admission	0.93 (0.82–1.06)	0.28	0.94 (0.83–1.07)	0.32	0.96 (0.83–1.12)	0.64	0.86 (0.75–0.98)	**0.03**
Male sex	0.91 (0.44–1.91)	0.81	1.12 (0.53–2.34)	0.76	0.99 (0.37–2.62)	0.98	1.35 (0.61–2.97)	0.45
Stroke	6.42 (2.08–18.89)	**0.001**	1.37 (0.48–3.93)	0.56	2.89 (0.85–9.85)	0.09	1.31 (0.39–4.38)	0.66
Heart failure	2.04 (1.10–3.82)	**0.03**	1.56 (0.84–2.90)	0.16	1.46 (0.65–3.27)	0.36	1.88 (0.98–3.60)	0.06
Myocardial ischemia/infarction	3.06 (0.73–12.87)	0.13	2.96 (0.71–12.40)	0.14	2.22 (0.29–16.74)	0.44	1.32 (0.18–9.71)	0.78
Hypertension	0.48 (0.26–0.88)	**0.02**	0.51 (0.28–0.93)	**0.03**	0.38 (0.18–0.82)	**0.01**	0.76 (0.38–1.54)	0.45
Renal insufficiency	0.47 (0.18–1.19)	0.11	0.52 (0.20–1.32)	0.17	0.57 (0.17–1.90)	0.36	0.52 (0.18–1.46)	0.21
Retinopathy	0.75 (0.40–1.41)	0.37	0.77 (0.41–1.43)	0.40	1.12 (0.52–2.43)	0.77	0.43 (0.21–0.89)	**0.02**
Steroids	0.75 (0.37–1.54)	0.44	0.995 (0.49–2.03)	0.99	1.26 (0.48–3.34)	0.64	1.81 (0.70–4.67)	0.22
Immunosuppressant	0.49 (0.15–1.59)	0.24	0.49 (0.15–1.60)	0.24	0.31 (0.04–2.29)	0.25	0.91 (0.32–2.59)	0.86
Antihypertensive agents	1.34 (0.67–2.66)	0.41	1.05 (0.54–2.04)	0.88	1.03 (0.43–2.43)	0.95	1.85 (0.77–4.43)	0.17
Antiplatelet drugs	0.52 (0.27–0.99)	**0.05**	0.61 (0.32–1.16)	0.13	0.44 (0.20–0.97)	**0.04**	0.60 (0.30–1.19)	0.14
Revascularization	0.44 (0.24–0.81)	**0.009**	0.45 (0.25–0.82)	**0.009**	0.28 (0.13–0.62)	**0.002**	0.47 (0.24–0.89)	**0.02**
Aortic regurgitation	1.45 (0.67–3.12)	0.34	1.36 (0.63–2.93)	0.43	1.37 (0.52–3.62)	0.53	1.08 (0.45–2.60)	0.86
Ascending aorta involvement	1.14 (0.48–2.70)	0.77	0.96 (0.40–2.29)	0.93	1.03 (0.35–3.01)	0.96	1.23 (0.54–2.82)	0.62
Aortic arch involvement	1.57 (0.73–3.41)	0.25	1.32 (0.61–2.85)	0.48	1.00 (0.34–2.92)	1.00	1.52 (0.70–3.33)	0.29
Descending aorta involvement	1.06 (0.56–1.98)	0.87	1.03 (0.55–1.91)	0.94	1.16 (0.53–2.53)	0.72	1.03 (0.52–2.02)	0.93
Abdominal aorta involvement	0.58 (0.31–1.10)	0.09	0.53 (0.29–0.99)	**0.05**	1.15 (0.54–2.46)	0.71	0.92 (0.49–1.75)	0.80
Renal artery involvement	0.55 (0.30–0.996)	**0.049**	0.43 (0.24–0.79)	**0.007**	0.40 (0.19–0.87)	**0.02**	0.48 (0.25–0.93)	**0.03**
Coronary artery involvement	3.05 (1.07–8.74)	**0.04**	3.16 (1.11–9.00)	**0.03**	2.73 (0.62–12.03)	0.18	1.65 (0.39–6.96)	0.50
Pulmonary artery involvement	1.51 (0.67–3.42)	0.32	1.55 (0.68–3.52)	0.29	1.32 (0.46–3.82)	0.61	1.99 (0.90–4.40)	0.09
Type IIb	1.73 (0.77–3.90)	0.19	2.03 (0.90–4.58)	0.09	3.13 (1.25–7.82)	**0.01**	1.37 (0.53–3.54)	0.52
Aneurysm	0.64 (0.23–1.79)	0.39	0.53 (0.19–1.49)	0.23	0.41 (0.10–1.76)	0.23	0.44 (0.13–1.42)	0.17
Vessel wall thickening	1.52 (0.77–3.00)	0.23	1.29 (0.66–2.51)	0.46	1.36 (0.59–3.14)	0.47	1.59 (0.82–3.09)	0.17
Elevated ESR	1.28 (0.68–2.43)	0.44	1.21 (0.64–2.29)	0.55	1.98 (0.92–4.31)	0.08	1.26 (0.64–2.47)	0.50
Elevated CRP	1.92 (1.01–3.64)	**0.047**	1.86 (1.00–3.47)	**0.05**	2.09 (0.95–4.58)	0.07	1.96 (1.02–3.79)	**0.04**

Significant *p* values are shown in bold typeface

*BMI* body mass index, *CI* confidence interval, *CRP* C-reactive protein, *ESR* erythrocyte sedimentation rate, *HR* hazard ratio

### Primary outcome: event-free survival

A total of 45 (44.6%) patients experienced at least one event at 1.05 (IQR 0.01–3.53) years after c-TA diagnosis. The 1-year, 3-year, 5-year, and 10-year event-free survival was 69.4%, 55%, 42.8%, and 27%, respectively. BMI (HR = 0.50, 95% CI 0.31–0.82, *p* = 0.006), heart failure (HR = 2.04, 95% CI 1.09–3.82, *p* = 0.026), stroke (HR = 6.42, 95% CI 2.08–18.89, *p* = 0.001), hypertension (HR = 0.48, 95% CI 0.26–0.88, *p* = 0.018), antiplatelet agents (HR = 0.52, 95% CI 0.27–0.99, *p* = 0.047), revascularization (HR = 0.44, 95% CI 0.24–0.81, *p* = 0.009), renal artery involvement (HR = 0.55, 95% CI 0.30–0.996, *p* = 0.049), coronary artery involvement (HR = 3.05, 95% CI 1.07–8.74, *p* = 0.038), and elevated CRP (HR = 1.92, 95% CI 1.01–3.64, *p* = 0.047) were associated with events in the univariate analysis. BMI (HR = 0.49, 95% CI 0.30–0.81, *p* = 0.005), stroke (HR = 7.37, 95% CI 2.35–23.11, *p* = 0.001), and revascularization (HR = 0.51, 95% CI 0.27–0.94, *p* = 0.032) were independent prognostic factors of events from the multivariate analysis.

### Complication-free survival

Vascular complications were observed in 45 (44.6%) patients at 1.5 (IQR 0.4–4) years after c-TA diagnosis. The 1-year, 3-year, 5-year, 10-year, and 15-year complication-free survival was 72.9%, 56.8%, 45.9%, 29.1%, and 19.4%, respectively. In the univariate model, associated factors included BMI (HR = 0.56, 95% CI 0.35–0.89, *p* = 0.015), hypertension (HR = 0.51, 95% CI 0.28–0.93, *p* = 0.028), revascularization (HR = 0.45, 95% CI 0.25–0.82, *p* = 0.009), abdominal aorta involvement (HR = 0.53, 95% CI 0.29–0.99, *p* = 0.047), renal artery involvement (HR = 0.43, 95% CI 0.24–0.79, *p* = 0.007), coronary artery involvement (HR = 3.16, 95% CI 1.11–9.01, *p* = 0.031), and elevated CRP (HR = 1.86, 95% CI 1.00–3.47, *p* = 0.05). In the multivariate model, BMI (HR = 0.58, 95% CI 0.37–0.93, *p* = 0.024) and renal artery involvement (HR = 0.47, 95% CI 0.25–0.86, *p* = 0.01) were statistically associated with vascular complications.

### Flare-free survival

At 1.7 (IQR 0.3–4.3) years of follow-up, the first disease flare was observed in 27 (26.7%) patients. The 1-year, 3-year, 5-year, 10-year, and 15-year flare-free survival was 85.3%, 71.1%, 62.3%, 54%, and 45%, respectively. BMI (HR = 0.44, 95% CI 0.23–0.84, *p* = 0.013), hypertension (HR = 0.38, 95% CI 0.18–0.82, *p* = 0.014), antiplatelet agents (HR = 0.44, 95% CI 0.20–0.97, *p* = 0.043), revascularization (HR = 0.28, 95% CI 0.13–0.62, *p* = 0.002), renal artery involvement (HR = 0.40, 95% CI 0.19–0.87, *p* = 0.022), and type IIb disease (HR = 3.13, 95% CI 1.25–7.82, *p* = 0.015) were associated with flares in the univariate model. Only revascularization (HR = 0.28, 95% CI 0.13–0.62, *p* = 0.002) was confirmed as a prognostic factor of flare in the multivariate model.

### Secondary outcome: rehospitalization-free survival

Thirty-eight (37.6%) patients rehospitalized within 1.7 (IQR 0.6–4.9) years after TA diagnosis. The 1-year, 3-year, 5-year, 10-year, and 15-year rehospitalization-free survival was 80.9%, 60.2%, 55.8%, 33.7%, and 25.3%, respectively. In the univariate analysis, age at admission (HR = 0.86, 95% CI 0.75–0.98, *p* = 0.027), retinopathy (HR = 0.43, 95% CI 0.21–0.89, *p* = 0.024), revascularization (HR = 0.47, 95% CI 0.24–0.89, *p* = 0.02), renal artery involvement (HR = 0.48, 95% CI 0.25–0.93, *p* = 0.029), and elevated CRP (HR = 1.96, 95% CI 1.02–3.79, *p* = 0.045) were significant prognostic factors for rehospitalization. In multivariate analysis, age at admission (HR = 0.81, 95% CI 0.69–0.94, *p* = 0.0055), renal artery involvement (HR = 0.49, 95% CI 0.25–0.96, *p* = 0.037), and elevated CRP (HR = 2.49, 95% CI 1.24–5.00, *p* = 0.011) were associated with rehospitalization.

### Era effect

The majority of children diagnosed with TA presented in the last decade of the study period. Additional file 1 (Table S3) compares the features of c-TA patients hospitalizing during the first (2002–2006, *n* = 27) and second (2007–2017, *n* = 74) periods. Children in the earlier period presented a higher proportion of catheter-based angiography (81.5% vs 56.8%, *p* = 0.022), limb claudication (37% vs 17.6%, *p* = 0.039), pulse deficits/decreases (59.3% vs 29.7%, *p* = 0.007), retinopathy (66.7% vs 27%, *p* = 0.00), and carotid artery involvement (59.3% vs 36.5%, *p* = 0.041), but a lower proportion of CTA operations (14.8% vs 71.6%, *p* = 0.00), abdominal aorta involvement (25.9% vs 48.6%, *p* = 0.04), vessel wall thickening (3.7% vs 37.8%, *p* = 0.001), and immunosuppressant prescription (0% vs 14.9%, *p* = 0.03). No significant difference was observed in terms of other demographic, clinical, imaging features, management, or outcomes and duration from admission to outcome onset.

## Discussion

To date this is the largest cohort describing the clinical profile and management of c-TA and the first study investigating the prognostic factors of rehospitalization and events including death, vascular complications, and disease flares in c-TA.

We observed that the majority of children endure TA in their second decade of life with 45.5% having over a 1-year delay to diagnosis and a near threefold higher prevalence in girls than boys, comparable to other c-TA cohorts [4–9]. Increasing age at first admission is an independent protective factor for rehospitalization/death in c-TA (*p* = 0.03), in accord with a contemporary study of 11 c-TA cases revealing a younger age at presentation (≤ 5 years) associated with higher mortality [5], while an increasing onset age in adults inclines to indicate refractory TA [19], given a prolonged and more severe disease course in younger children and increased cardiovascular risk factors in elder adults. We also observed 40.6% of children with BMI < 18.5 kg/m^2^, and elevated BMI indicates decreased further events (*p* = 0.005) and vascular complications (*p* = 0.024), corresponding to Liu et al. [19] who analyzed 262 adult TA cases and confirmed low BMI as an independent factor of increased previous/present cardiovascular diseases [19]. However, the underlying mechanisms are unclear with a possible explanation of muscle mass loss in chronic disease conditions relating to physical disability and functional impairment.

The spectrum of clinical features in c-TA are heterogeneous. Arterial hypertension, asymmetric blood pressure, bruits, and pulse deficits are the core manifestations, in line with a EULAR/PRINTO/PReS study analyzing the clinical phenotypes of 87 c-TA patients (Asian, *n* = 9) [11]. Hypertension, a representative phenotype of TA with a published higher prevalence of 56–100% in children than in adults(33–83%) [4–9, 18, 20], is observed in 70.3% of our c-TA patients as a protective indicator of vascular sequela and disease flare from univariate analysis (*p* < 0.05). Causes of hypertension comprise renal artery stenosis (RAS; 78.9%, *n* = 56), mid-aortic stenosis (MAS; defined as thoracic aorta and/or abdominal aorta stenosis, 45.0%, *n* = 32), and severe aortic regurgitation (2.8%, *n* = 2). Local lesions affecting renal arteries or the abdominal aorta (type IV) dominate in c-TA, unlike adult TA which is characterized with the most extensive lesion from the ascending aorta to the abdominal aorta indicating unfavorable outcomes (type V) [18]. Goel et al. [20] report type IV in TA, predicting a 2.2-fold probability of persistent complete steroid response and a feasible reduced dose to ≤ 5 mg/day, while abdominal aorta involvement is an independent factor for decreased steroid-refractory TA [20]. Remarkably, in c-TA, we firstly identify renal artery involvement as an independent prognostic factor of fewer vascular complications (*p* = 0.015) and rehospitalization (*p* = 0.04), while those with an abdominal aorta lesion show a trend for less frequent vascular complications. Furthermore, type IIb—the lesion from the ascending to the thoracic aorta except for renal artery or abdominal aorta involvement—was analyzed as a 3.1-fold risk predictor of c-TA relapse and indicates a trend for vascular complications. Thus, renal artery or abdominal aorta involvement, particularly in those presenting with hypertension, predicts relatively satisfactory outcomes in c-TA for a better steroid response, limited recurrent inflammation, and decreased vascular complications and re-admission.

The differential diagnosis of c-TA from other diseases with similar manifestations is critical but challenging [14, 21]. Childhood FMD, often presenting with isolated RAS or confined peri-renal aortic stenosis rather than the classic “string of beads” phenotype, also presents a differential disease with typical lesions in the middle or distal segment of medium-sized arteries [22]. Inflammation, however, serves as an exclusive and prognostic phenotype of TA. Comarmond et al. analyzed 318 TA patients with a 6-year follow-up, identifying CRP ≥ 7 mg/L as independent risk factor of relapse by twofold [18], and Goel et al. confirmed CRP < 6.2 mg/L as an independent positive factor for sustained inactive disease in 251 TA patients [20]. Notably, we firstly identify elevated CRP (≥ 6 mg/L) as independent 2.5-fold predictor of re-admission (*p* = 0.01) in the c-TA population, and a potential risk factor of vascular complications or re-flares approaching twofold.

In terms of therapy, the initial goals are to suppress inflammation and to prevent/release future vascular ischemia, but evidence-based recommendations in c-TA are limited. GCs remain the mainstay in 78.2% of our c-TA cohort for inflammation control or revascularization preparation with a median initial dose of 0.5 (IQR 0.4–0.6) mg/kg/day, corresponding to a recent Indian study revealing comparable complete remission rates and future complication risk between arms under baseline prednisone 0.5 mg/kg/day and 1 mg/kg/day, adjusting for CRP, ESR, juvenile onset type, and type IV disease [20]. Additional antihypertensive agents are prescribed in 72.3% with calcium channel blockers and/or β-blockers preferred in children based on blood pressure level, cardiac function, and renal function. Antiplatelet drugs are used in 72.3% for thrombosis prevention in conditions of revascularization and hypercoagulable state due to inflammation or prednisone administration, with a trend for isolated low-dose aspirin prescription in c-TA. De Souza et al., observing 48 adult TA patients during a 6.4-year follow-up, revealed antiplatelet therapy as a predictor of decreased acute ischemic events with few bleeding outcomes [23]. Similarly, we observe antiplatelet drugs predicting around 0.5-fold further events from univariate analysis, indicating the potential role of antiplatelet drugs in the secondary prevention of c-TA patients.

Arterial reconstruction in c-TA is necessary for end-organ or limb ischemia reversion. We observe 57.4% of c-TA patients undergoing revascularizations with endovascular approaches being increasingly implied, especially in affected renal artery, subclavian artery, or mid-aorta. Strikingly, revascularization in our cohort is firstly shown to be an independent protective factor of fewer events and flares in c-TA by 0.51-fold and 0.28-fold. Feasible explanations of decreased events and flares include: 1) revascularization helps major complication amelioration and consequent prognosis and life expectancy improvement [24]; 2) prednisone is generally administered in c-TA patients at least 2 months prior to and 6 months after procedures, even in patients at a clinical quiescent stage for potential active TA at a subclinical level [17] and restenosis or re-intervention prevention [12, 15]; and 3) appropriate perioperative administration of antiplatelet agents as above. Additionally, the correlation between revascularization, hypertension, and renal artery involvement is statistically significant (chi-squared test, *p* < 0.05), with the latter two also analyzed as optimistic predictors of c-TA outcomes. Convincing data are revealed regarding the promising role of revascularization on RAS in TA. Hong et al. [25] observed an inclination to perform procedures in younger RAS patients caused by TA without risk for further chronic renal insufficiency, refractory hypertension, and/or death, while Ladapo et al. [26] reported nearly 50% of hypertensive c-TA patients achieving a blood pressure benefit after revascularization in RAS. Thus, revascularization with adequate perioperative immunosuppressive and antiplatelet therapy is beneficial in c-TA, particularly in hypertensive patients with renal artery involvement.

Regarding prognosis, early mortality for c-TA (3%) is observed in the first year after discharge, while around 50% c-TA patients endure an event or rehospitalization within first 5 years following diagnosis, astonishingly 5 years earlier than in adult TA [18]. Two patients died by 1 month of recurrent acute heart failure, while one died from severe three-vessel coronary artery involvement. From the univariate analysis, heart failure (24.8%) is a 2.04-fold risk factor of events with a tendency for rehospitalization, while coronary artery involvement observed in only 5% of c-TA patients predicts a greater than threefold risk of events and vascular complications (*p* < 0.05). Early heart failure reversion and coronary artery lesion amelioration are therefore urgently needed, with prompt revascularization recommended, particularly for acute heart failure reversion and CABG after inflammation control [14, 27]. Furthermore, stroke at onset independently indicates a 7.37-fold risk of future events in our c-TA patients (*p* = 0.032). A French study revealed that, among TA patients with stroke, neurological impairment and recurrent stroke are observed in 59% and 35% at a median 137-month follow-up. Although there is limited evidence on stroke management, low-dose aspirin, immunosuppressive therapy, and reconstructive procedures are commonly administered, while 24% of strokes occur after surgery and 35% require further carotid artery intervention [28]. Similarly, a recent meta-analysis of 770 TA patients with 1363 lesions shows strokes occurring less with intervention compared with surgery (odds ratio = 0.33, *p* = 0.003) [29]. Therefore, stroke, heart failure, or coronary artery involvement at initial presentation alert us to a high risk of further events in c-TA patients, requiring close monitoring and relatively aggressive management, with a promising role for CABG in coronary artery lesions and interventions in acute heart failure and stroke management, while long-term, large-scale studies are required to address these issues.

Our study has several limitations. We acknowledge that the majority of patients were recruited in a retrospective cohort before December 2016 with all the associated confounding factors, whereas we initiated prospective enrollment from January 2017 for comprehensive longitudinal data collection. The sample size of c-TA is small, although it does represent the largest one worldwide. Furthermore, the study spans nearly 16 years (January 2002 to December 2017), which indicates changes in clinical diagnosis and management of TA and their consequent contributions to outcomes. Notably, variables in our Cox model are not statistically correlated with time after the Schoenfeld’s residuals test and no significant differences in terms of outcomes are observed after intergroup comparison of patients enrolled prior to and after 2007. Finally, our patients transferred from multiple centers/regions over the country, and were recruited in a tertiary referral center with a higher potential for severe disease spectrum and referral bias.

## Conclusions

This large ambispective study of c-TA reveals an early mortality and morbidity, with 3% of patients dying by the first year and around 50% enduring at least an event or rehospitalization within the first 5 years after diagnosis. Hypertension, renal artery involvement, and revascularization based on glucocorticoids, antihypertensive drugs, and antiplatelet agents are the core clinical, imaging, and therapeutic features of c-TA with optimistic indications on further prognosis. Stroke, elevated CRP, lower BMI level, and younger age at admission are, however, independent risk factor of poor outcomes. Further studies are required to construct a risk assessment model of c-TA and its validation.

## Additional file

Additional file 1: **Table S1.** Comparison of reported cohorts on c-TA after 2010 (data before 2010 are summarized by Brunner et al. [4]). **Table S2.** Clinical presentations of c-TA categorized by organic systems. **Table S3.** Comparison of demographic, clinical, laboratory, imaging, therapeutic features, and outcomes between c-TA patients hospitalized before and after 2007. (DOCX 27 kb)

## Abbreviations

ACR: American College of Rheumatology
ARP: Acute-phase reactant
BMI: Body mass index
CABG: Coronary artery bypass graft
CI: Confidence interval
CRP: C-reactive protein
c-TA: Childhood Takayasu’s arteritis
CTA: Computed tomographic angiography
ESR: Erythrocyte sedimentation rate
EULAR: European League against Rheumatism
FDG-PET: Fluorodeoxyglucose positron emission tomography
FMD: Fibromuscular dysplasia
GC: Glucocorticoid
HR: Hazard ratio
IQR: Interquartile range
MMF: Mycophenolate mofetil
MRA: Magnet resonance angiography
NIH: National Institutes of Health
PreS: Pediatric Rheumatology European Society
PRINTO: Pediatric Rheumatology International Trials Organization
RAS: Renal artery stenosis
SUVmax: Maximum standard uptake value
TA: Takayasu’s arteritis
