# Correction to: Clinical course and prognostic factors of childhood Takayasu’s arteritis: over 15-year comprehensive analysis of 101 patients

**DOI:** 10.1186/s13075-019-1844-8

**Published:** 2019-02-14

**Authors:** Luyun Fan, Huimin Zhang, Jun Cai, Lirui Yang, Bin Liu, Dongmei Wei, Jiachen Yu, Jiali Fan, Lei Song, Wenjun Ma, Xianliang Zhou, Haiying Wu, Ying Lou

**Affiliations:** 1 State Key Laboratory of Cardiovascular Disease, National Center for Cardiovascular Diseases, Fuwai Hospital, Chinese Academy of Medical Sciences and Peking Union Medical College, Beijing, China; 2 Department of Cardiology, Beijing Anzhen Hospital, Capital Medical University, Beijing, China; 3 School of Basic Medicine, Chinese Academy of Medical Sciences and Peking Union Medical College, Beijing, China

**Correction to: Arthritis Res Ther**

**https://doi.org/10.1186/s13075-018-1790-x**

Following publication of the original article [1], the authors reported an error. “Angioplasty” in “Imaging modalities” of the “Methods” section should be replaced by “Angiography”.

The sentence should read: Angiography was performed in all c-TA patients, including conventional angiography, CTA, and/or magnet resonance angiography (MRA) as the initial diagnostic modality in 36 (35.6%), 57 (56.4%), and 9 (8.9%), respectively, while 28 (27.7%) patients experienced additional catheter-based angiography for interventional therapy after TA diagnosis by CTA (24.7%, *n* = 25) and/or MRA (4%, *n* = 4).
