# Supplementary material for: Clinical course and prognostic factors of childhood Takayasu’s arteritis: over 15-year comprehensive analysis of 101 patients
Source: Arthritis Res Ther. 2019 Jan 22;21:31. doi: 10.1186/s13075-018-1790-x (PMC6341556; doi:10.1186/s13075-018-1790-x)
Supplement: Supplementary file 1 — Table S1. Comparison of reported cohorts on c-TA after 2010 (data before 2010 are summarized by Brunner et al. [4]). Table S2. Clinical presentations of c-TA categorized by organic systems. Table S3. Comparison of demographic, clinical, laboratory, imaging, therapeutic features, and outcomes between c-TA patients hospitalized before and after 2007. (DOCX 27 kb) [file 13075_2018_1790_MOESM1_ESM.docx]

**Additional file 1**

**Table S1. Comparison of reported cohorts on c-TA after 2010(Data before 2010 are summarized by Brunner et al.[4]).**

| Study | Florence | Feng | Eleftheriou | Misra | Clemente | Szugye | Goel | Zhu | Jales-Neto |
| --- | --- | --- | --- | --- | --- | --- | --- | --- | --- |
| Year | 2017 | 2017 | 2015 | 2015 | 2014 | 2014 | 2014 | 2010 | 2010 |
| Location | Canada | China | UK | India | Brazil | USA | South India | China | Brazil |
| Number of patients | 27 | 11 | 11 | 29 | 71 | 21 | 40 | 14 | 17 |
| Sex ratio(female to male） | 20:7 | 7:4 | 7:4 | 19:10 | 51:20 | 15:6 | 26:14 | 11:3 | 11:6 |
| Median age at TA onset, years | NA | 9.4 | NA | 13 | 9.2* | NA | 12.5 | NA | 16 |
| Median age at TA diagnosis, years | 12.4 | NA | 11.8 | 14 | NA | 13 | 13.5 | 10.2 | NA |
| Median delay to diagnosis, months | 6 | 1.4 | NA | 12 | 14.4* | 6 | 11.3 | 1.8 | 36 |
| Median duration of follow-up, years | 2.1 | NA | 1.3 | 2.4 | 5.4* | 1.2 | 1.1 | 3 | 8.37* |
| Flare,% | 48 | NA | NA | 20 | NA | NA | 37.5 | 14.3 | 23.5 |
| Mortality,% | 7 | NA | 27 | 3 | 7 | 0 | 3 | 0 | 11.8 |
| Glucocorticoids,% | 82 | 100 | 81 | 80 | 90 | 86 | 85 | NA | 76 |
| Immunosuppressive drugs,% | 67 | 9 | 9 | 85 | NA | 81 | 85 | NA | NA |
| Surgeries/Interventions,% | 30 | 18 | 45 | 20 | 63 | 38 | 90 | 35.7 | 52.9 |
| Hypertension,% | 56 | 1 | 73 | 76 | 85 | 57 | 73 | 93 | 64.7 |
| Heart failure,% | NA | 9 | 18 | 14 | 18.3 | NA | NA | 29 | 17.6 |
| Myocardial infraction/angina pectoris,% | NA | NA | 18 | NA | NA | NA | NA | NA | 5.8 |
| Stroke,% | 11 | NA | 18 | 7 | NA | NA | 8 | 0 | 17.6 |
| Claudication,% | 22 | NA | 9 | 41 | 36.6 | 0 | 40 | 29 | 58.8 |
| Pulse loss/deficits,% | 59 | 27.2 | 18 | 79 | 85.9 | 61.9 | 63 | NA | 59 |
| Bruits,% | 56 | 27.2 | 45 | 48 | 74.6 | 57.1 | 47 | NA | 58.8 |
| Fever,% | 19 | 45.4 | 36 | 55 | NA | 14.3 | 45 | 29 | 41.2 |
| Elevated ESR,% | NA | 90 | NA | 79 | 81 | 100 | 65.7 | 64 | NA |

NA: Data are not reported; *: mean.

**Table S2. Clinical presentations of c-TA categorized by organic systems.**

| Symptoms/Signs | Patients | Percentage, % |
| --- | --- | --- |
| Cardiovascular system | 99 | 98 |
| Hypertension | 71 | 70.3 |
| Blood pressure discrepancy | 56 | 55.4 |
| Bruits | 52 | 51.5 |
| Pulse deficits | 38 | 37.6 |
| Heart failure | 25 | 24.8 |
| Claudication | 23 | 22.8 |
| Palpitation | 9 | 8.9 |
| Angina pectoris | 2 | 2 |
| Myocardial infarction | 1 | 1 |
| Neurological/Ocular system | 45 | 44.6 |
| Dizziness | 27 | 26.7 |
| Syncope | 10 | 9.9 |
| Blurred vision | 8 | 7.9 |
| Stroke | 6 | 5.9 |
| TIA | 2 | 2 |
| Headache | 1 | 1 |
| Amaurosis | 1 | 1 |
| Pulmonary system | 34 | 33.7 |
| Dyspnea | 30 | 29.7 |
| Cough | 12 | 11.9 |
| Chest pain | 2 | 2 |
| Hemoptysis | 1 | 1 |
| Gastrointestinal system | 4 | 4 |
| Abdominal Pain | 4 | 4 |
| Constitutional system | 30 | 29.7 |
| Fever | 13 | 12.9 |
| Anorexia | 11 | 10.9 |
| Malaise | 9 | 8.9 |
| Weight loss | 4 | 4 |
| Carotidynia | 4 | 4 |
| Musculoskeletal system | 5 | 5 |
| Myalgia | 3 | 3 |
| Arthritis/Arthralgia | 2 | 2 |

**Table S3. Comparison of demographic, clinical, laboratory, imaging, therapeutic features and outcomes of c-TA patients hospitalized before and after 2007.**

|  | Patients enrolled before 2007(n=27) | Patients enrolled after 2007(n=74) | p value |
| --- | --- | --- | --- |
| **Demographic features** | Median[IQR] | Median[IQR] |  |
| Age at TA admission, years | 15 [13,16] | 16[14,17.3] | 0.051 |
| Male sex, n(%) | 9(33.3) | 15(20.3) | 0.17 |
| Delay to diagnosis, years | 0.54[0.17,3.08] | 0.94[0.17,2.02] | 0.76 |
| BMI, kg/m^2^ | 19.8[16.4,22] | 19.6[17.8,21.3] | 0.50 |
| **Clinical presentations** | n(%) | n(%) |  |
| Stroke | 3(11.1) | 3(4.1) | 0.34 |
| Heart failure | 4(14.8) | 21(28.4) | 0.16 |
| Myocardial ischemia/infraction | 0(0) | 3(4.1) | 0.56 |
| Claudication | 10(37) | 13(17.6) | **0.04** |
| Hypertension | 18(66.7) | 53(71.6) | 0.63 |
| Systematic sypmtoms | 10(37) | 20(27) | 0.33 |
| Bruits | 14(51.9) | 38(51.4) | 0.96 |
| Pulse deficits/decreases | 16(59.3) | 22(29.7) | **0.007** |
| Retinopathy | 18(66.7) | 20(27) | **0.00** |
| **Management** | n(%) | n(%) |  |
| Glucocorticoids | 18(66.7) | 61(82.4) | 0.09 |
| Immunosuppressant | 0(0) | 11(14.9) | **0.03** |
| Antihypertensive agents | 19(70.4) | 54(73) | 0.80 |
| Antiplatelet drugs | 23(85.2) | 50(67.6) | 0.08 |
| Statins | 0(0) | 5(6.8) | 0.17 |
| Revascularization | 16(59.3) | 42(56.8) | 0.82 |
| Intervention | 16(59.3) | 39(52.7) | 0.56 |
| Surgery | 2(7.4) | 6(8.1) | 0.91 |
| **Imaging modalities** | n(%) | n(%) |  |
| CTA | 4(14.8) | 53(71.6) | **0.00** |
| MRA | 5(18.5) | 7(9.5) | 0.21 |
| Catheter-based angioplasty | 22(81.5) | 42(56.8) | **0.02** |
| 18F-FDG-PET/CT | 0 | 7(9.5) | 0.10 |
| **Vessel beds involvement** | n(%) | n(%) |  |
| Aortic Regurgitation | 2(7.4) | 12(16.2) | 0.26 |
| Ascending aorta | 4(14.8) | 9(12.2) | 0.73 |
| Aortic arch | 4(14.8) | 13(17.6) | 0.74 |
| Thorcic descending aorta | 6(22.2) | 27(36.5) | 0.18 |
| Abdominal descending aorta | 7(25.9) | 36(48.6) | **0.04** |
| Carotid artery | 16(59.3) | 27(36.5) | **0.04** |
| Subclavian artery | 15(55.6) | 29(39.2) | 0.14 |
| Renal artery | 17(16.8) | 46(62.2) | 0.94 |
| Iliac artery | 2(7.4) | 11(14.9) | 0.21 |
| Coronary artery | 0 | 5(6.8) | 0.32 |
| Pulmonary artery | 1(3.7) | 11(14.9) | 0.17 |
| **Type of lesions** | n(%) | n(%) |  |
| Stenosis | 25(92.6) | 67(90.5) | 0.75 |
| Aneurysm | 4(14.8) | 8(10.8) | 0.73 |
| Occlusion | 14(51.9) | 50(67.6) | 0.15 |
| Vessel wall thickening | 1(3.7) | 28(37.8) | **0.001** |
| **Hata and Numanio's classification** | n(%) | n(%) |  |
| Type I disease | 5(18.5) | 7(9.5) | 0.30 |
| Type IIb disease | 4(14.8) | 6(8.1) | 0.45 |
| Type III disease | 0 | 9(12.2) | 0.11 |
| Type IV disease | 9(33.3) | 29(39.2) | 0.59 |
| Type V disease | 9(33.3) | 23(31.1) | 0.83 |
| **Disease Activity** | 16(59.3) | 45(60.8) | 0.89 |
| Elevated ESR | 9(33.3) | 24(32.4) | 0.93 |
| Elevated CRP | 6(22.2) | 29(39.2) | 0.11 |
| **Outcomes** | n(%) | n(%) |  |
| Death | 0 | 3(4.1) | 0.29 |
| Events | 12(44.4) | 33(44.6) | 0.99 |
| Re-hospitalization | 8(29.6) | 30(40.5) | 0.32 |
| Vascular Complications | 12(44.4) | 33(44.6) | 0.99 |
| Re-flares | 9(33.3) | 18(24.3) | 0.37 |

BMI: body mass index; ESR: erythrocyte sedimentation rate; CRP, C-reactive protein; TA: Takayasu arteritis; IQR: interquartile range; CTA: computed tomographic angiography; MRA: magnet resonance angiography; 18F-FDG-PET: 18F-fluorodeoxyglucose positron emission tomography.
